# Prolactin stimulates the proliferation of normal female cholangiocytes by differential regulation of Ca^2+^-dependent PKC isoforms

**DOI:** 10.1186/1472-6793-7-6

**Published:** 2007-07-19

**Authors:** Silvia Taffetani, Shannon Glaser, Heather Francis, Sharon DeMorrow, Yoshiyuki Ueno, Domenico Alvaro, Luca Marucci, Marco Marzioni, Giammarco Fava, Julie Venter, Shelley Vaculin, Bradley Vaculin, Ian Pak-Yan Lam, Vien Hoi-Yi Lee, Eugenio Gaudio, Guido Carpino, Antonio Benedetti, Gianfranco Alpini

**Affiliations:** 1 Department of Gastroenterology, Università Politecnica delle Marche, Nuovo Polo Didattico, III piano, Via Tronto 10 Ancona, 60020, Italy; 2 Department of Medicine, Scott & White Hospital and Texas A&M University System Health Science Center, College of Medicine, MRB, 702 SW H.K. Dodgen Loop, Temple, TX, 76504, USA; 3 Division of Research and Education, Scott & White Hospital and Texas A&M University System Health Science Center, College of Medicine, MRB, 702 SW H.K. Dodgen Loop, Temple, TX, 76504, USA; 4 Division of Gastroenterology, Tohoku University School of Medicine, 1-1 Seiryo, Aobaku, Sendai 980-8574, Japan; 5 Division of Gastroenterology, University of Rome, "La Sapienza", via Roberto Rossellini 51, 00137 Rome, Italy; 6 Division of Research, Central Texas Veterans Health Care System, MRB, 702 SW H.K. Dodgen Loop, Temple, TX, 76504, USA; 7 School of Biological Sciences, The University of Hong Kong, Pokfulam Road, Hong Kong, China; 8 Department of Human Anatomy, University of Rome, "La Sapienza", Via Alfonso Borelli 50, 00161, Rome, Italy; 9 Department of Systems Biology and Translational Medicine, Texas A&M University System Health Science Center, College of Medicine, MRB, 702 SW H.K. Dodgen Loop, Temple, TX, 76504, USA

## Abstract

**Background:**

Prolactin promotes proliferation of several cells. Prolactin receptor exists as two isoforms: long and short, which activate different transduction pathways including the Ca^2+^-dependent PKC-signaling. No information exists on the role of prolactin in the regulation of the growth of female cholangiocytes. The rationale for using cholangiocytes from female rats is based on the fact that women are preferentially affected by specific cholangiopathies including primary biliary cirrhosis. We propose to evaluate the role and mechanisms of action by which prolactin regulates the growth of female cholangiocytes.

**Results:**

Normal cholangiocytes express both isoforms (long and short) of prolactin receptors, whose expression increased following BDL. The administration of prolactin to normal female rats increased cholangiocyte proliferation. In purified normal female cholangiocytes, prolactin stimulated cholangiocyte proliferation, which was associated with increased [Ca^2+^]_i _levels and PKCβ-I phosphorylation but decreased PKCα phosphorylation. Administration of an anti-prolactin antibody to BDL female rats decreased cholangiocyte proliferation. Normal female cholangiocytes express and secrete prolactin, which was increased in BDL rats. The data show that prolactin stimulates normal cholangiocyte growth by an autocrine mechanism involving phosphorylation of PKCβ-I and dephosphorylation of PKCα.

**Conclusion:**

We suggest that in female rats: (i) prolactin has a trophic effect on the growth of normal cholangiocytes by phosphorylation of PKCβ-I and dephosphorylation of PKCα; and (iii) cholangiocytes express and secrete prolactin, which by an autocrine mechanism participate in regulation of cholangiocyte proliferation. Prolactin may be an important therapeutic approach for the management of cholangiopathies affecting female patients.

## Background

Cholangiocytes have a low replicative activity in the normal state [1-3], but they proliferate or undergo apoptosis in cholangiopathies [3-6], progressive liver disorders characterized by an abnormal balance between cholangiocyte proliferation and death, leading to vanishing of intrahepatic bile ducts [3,4]. It has been hypothesized that sex hormones play a role in the pathogenesis of some cholangiopathies [4,7,8]. In particular, the most common of them, primary biliary cirrhosis (PBC), is more common in women, and its clinical outbreak is typically after menopause [4,9]. The low expression of estrogen receptor alpha in PBC and their disappearance in the advanced histological stages of this disease suggests that an estrogenic deficiency could favor the evolution of PBC toward ductopenia [7]. Furthermore, a study demonstrated that: (i) ovariectomy to BDL female rats induced a decrease in intrahepatic ductal mass; and (ii) administration of 17-β estradiol during BDL to ovariectomized rats prevented the decrease in the number of bile ducts [10].

Prolactin is a pituitary hormone and a pleiotropic cytokine that promotes cellular proliferation, differentiation and survival in a number of cells [11]. Two different isoforms of the prolactin receptor exist: they are both encoded by a single gene, by which the two isoforms (a short and a long form) are obtained by alternative splicing [12]. The long and short forms are both membrane bound receptors with an identical binding site for prolactin, but differ in the length of their cytoplasmic tail [12]. Prolactin binding to the long or short form of prolactin receptors activates different signaling pathways including mitogen-activated protein kinase (MAPK) [13], JAK/STAT [14], and Ca^2+^/PKC [13]. While long prolactin receptors activate several signaling pathways including JAK/STAT [15], the short isoform of prolactin receptor activates various kinases and interacts with 17-hydroxy-steroid dehydrogenase pathways [16,17]. The long form of the prolactin receptor mediates activation of the Ca^2+^-dependent PKC signaling in a number of cells [18,19].

Although studies have shown differences in the expression of prolactin receptors between hepatocytes and cholangiocytes of normal and cholestatic livers [20-23], no information exists on the role of prolactin on the regulation of cholangiocyte growth. The rationale for using cholangiocytes from female rats is based on the fact that women are preferentially affected by specific cholestatic liver diseases including PBC [9]. We addressed these questions: (i) Do normal and BDL female and male cholangiocytes express prolactin receptors? (ii) Does *in vivo *administration of prolactin to normal female and male rats increase cholangiocyte proliferation? (iii) Are prolactin effects on normal cholangiocyte proliferation of female rats associated with increased intracellular Ca^2+ ^([Ca^2+^]_i_) levels and differential phosphorylation of Ca^2+^-dependent PKC isoforms (α, β-I, β-II and γ, which are important in the regulation of biliary functions) [24-29]? (iv) Does the *in vivo *administration of an anti-prolactin antibody to BDL female and male rats inhibit cholangiocyte hyperplasia? and (v) Do female cholangiocytes express the message and protein for prolactin and secrete prolactin?

## Results

### Cholangiocytes express prolactin receptors

**Figure 1:**
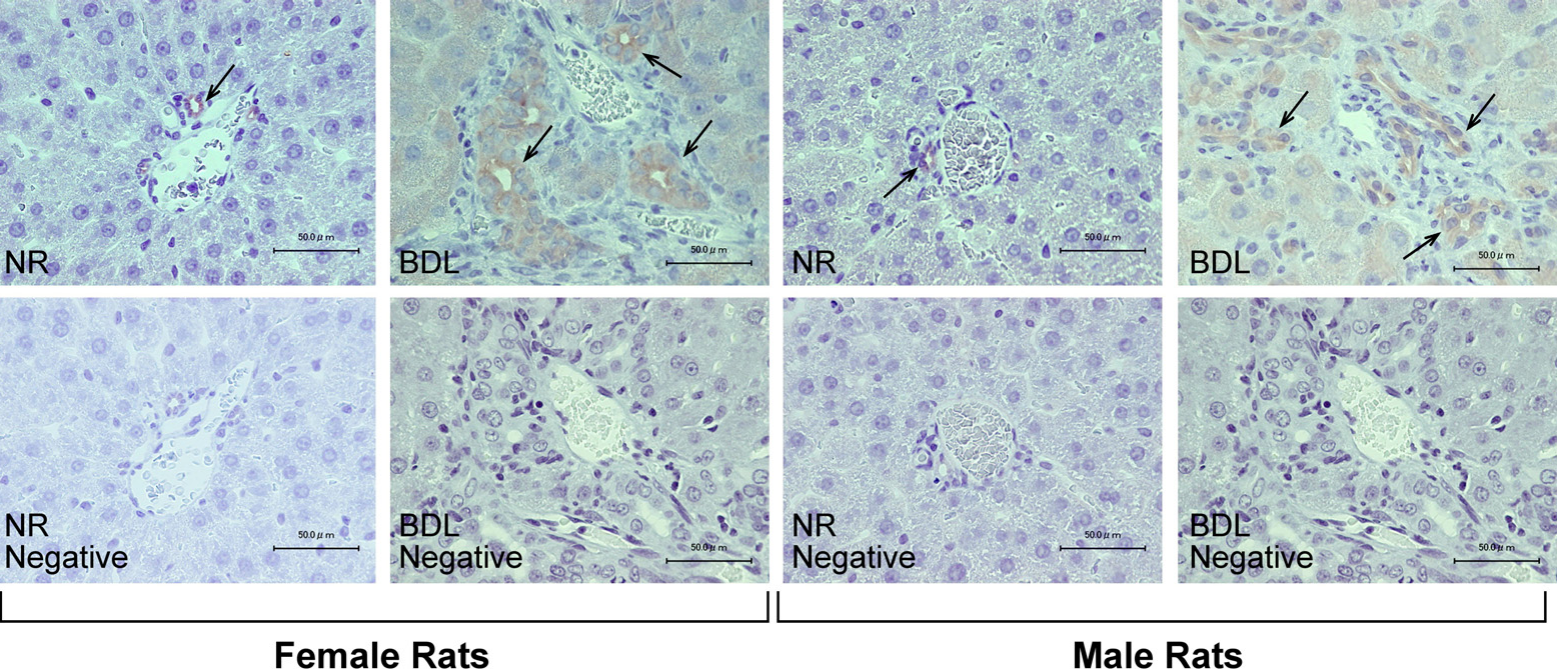
The localization of prolactin receptor in the liver was evaluated by immunohistochemistry (scale bar = 50 μm) in liver sections from normal and BDL female and male rats. Bile ducts from normal and BDL female and male rats express these receptors (arrows). No immunohistochemical reaction was observed when a consecutive liver section of the same field was incubated with non-immune serum. Hepatocytes from normal and BDL female and male rats express the prolactin receptor.

**Figure 2:**
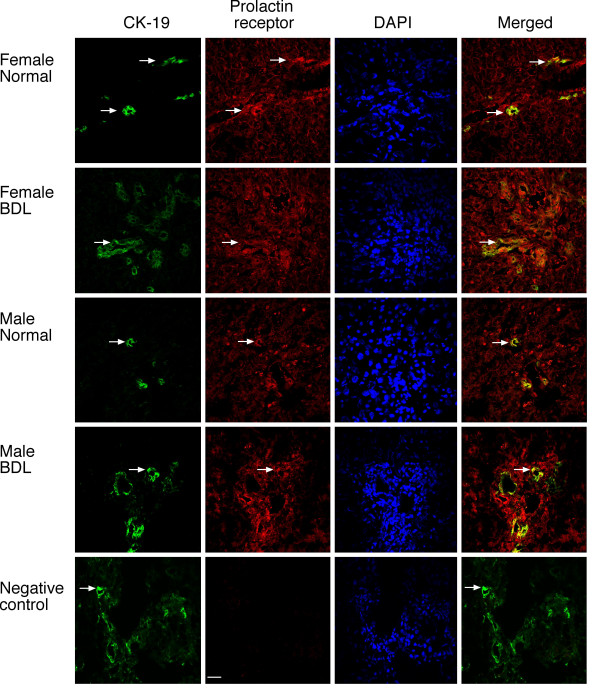
The localization of prolactin receptor in the liver was evaluated by immunofluorescence (scale bar = 20 μm) in liver sections from normal and BDL female and male rats. By immunofluorescence, prolactin receptor immunoreactivity (red) was co-localized with CK-19 immunoreactivity (green; indicated by arrows) demonstrating cholangiocyte expression; sections were counterstained with DAPI; in the merged photograph we show co-localization of prolactin receptor and CK-19. Hepatocytes from normal and BDL female and male rats express the prolactin receptor.

Immunohistochemistry in liver sections from normal and BDL female and male rats shows that cholangiocytes express prolactin receptors (Figure 1, see arrows). By immunofluorescence, immunoreactivity for prolactin receptor is co-localized with the expression of cytokeratin-19 (CK-19, a marker of cholangiocytes) [2] (Figure 2); in the merged photograph there is co-localization of prolactin receptor and CK-19 (Figure 2). No immunohistochemical reaction was observed when a consecutive liver section of the same field was incubated with non-immune serum (Figure 1). Parallel to other studies [23], prolactin receptors are also expressed by hepatocytes from normal and BDL female and male rats (Figures 1 and 2).

**Figure 3:**
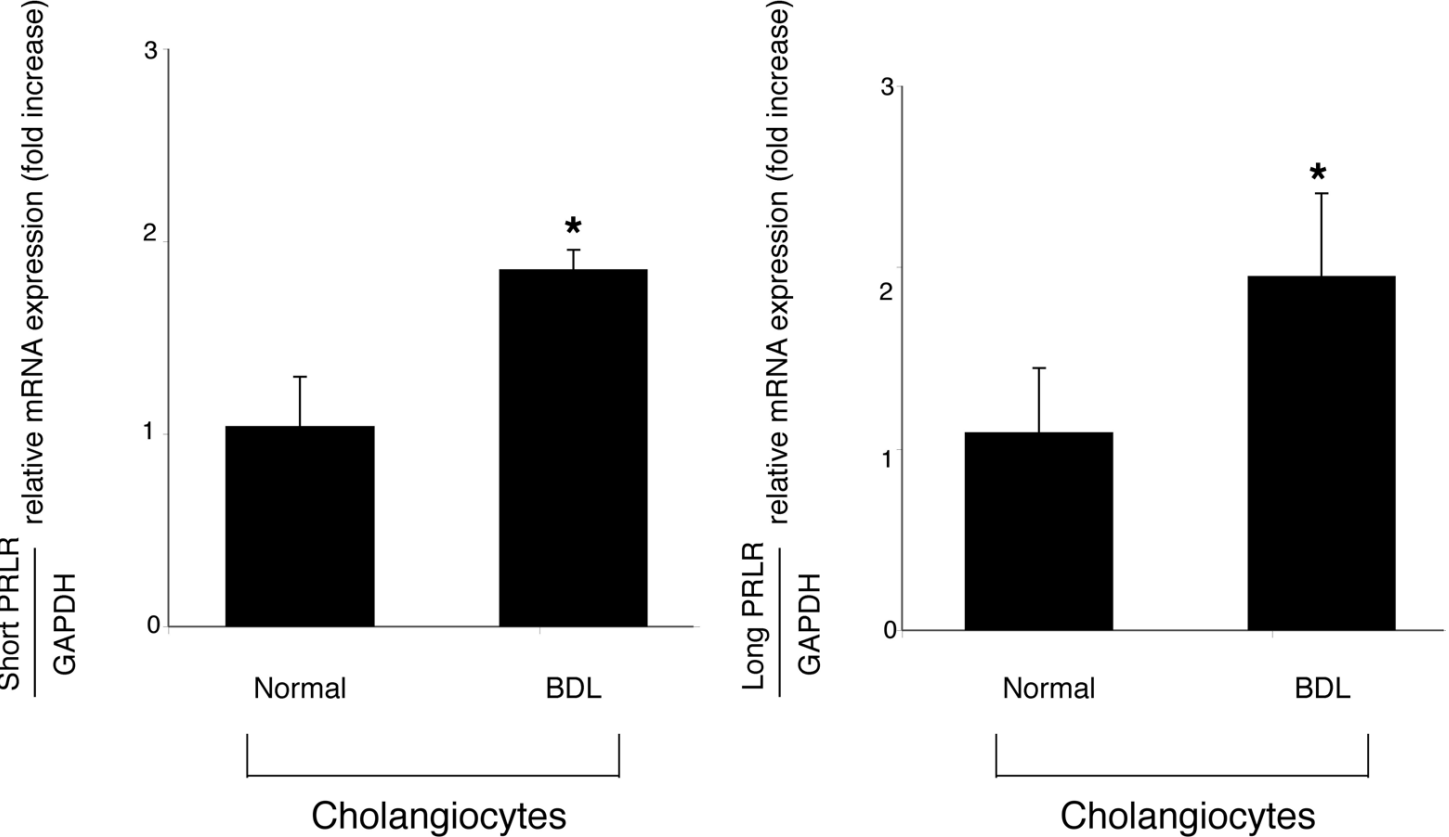
Real time PCR for the message for the short and long form of prolactin receptor in total cholangiocyte RNA (0.75 μg) from normal and BDL female rats. Normal female cholangiocytes express both the short and long form of prolactin receptor mRNA (expressed as ratio to GAPDH mRNA); following BDL, the expression of both the short and long form of prolactin receptor mRNA (expressed as ratio to GAPDH mRNA) significantly increased in purified cholangiocytes. Data are mean ± SEM of 3 experiments. *p < 0.05 vs. relative expression of short and long prolactin receptor mRNA of normal cholangiocytes. NR = normal rat; PRLR = prolactin receptor.

By RT-PCR, normal and BDL female cholangiocytes expressed the expected molecular weight band for the message for the short (582 bp) and long (781 bp) form of prolactin receptor, and for glyceraldehyde-3-phosphate dehydrogenase (GAPDH, the housekeeping gene) (294 bp) (not shown). Sequence analysis of the RT-PCR fragments shows that both the rat short and long prolactin receptors are 98% homologous to the short (NCBI Genbank accession No. NM 012630) [23] and long rat prolactin receptor mRNAs (NCBI Genbank accession No. NM 001034111) [23]. By real time PCR, normal female cholangiocytes express both the short and long form of prolactin receptor mRNA (expressed as a ratio to GAPDH mRNA) (Figure 3); following BDL, the expression of the short and long form of prolactin receptor mRNA significantly increased in purified female cholangiocytes (Figure 3).

### Effect of *in vivo *administration of prolactin to normal rats on serum prolactin levels, portal inflammation, necrosis and cholangiocyte apoptosis and proliferation

**Figure 4:**
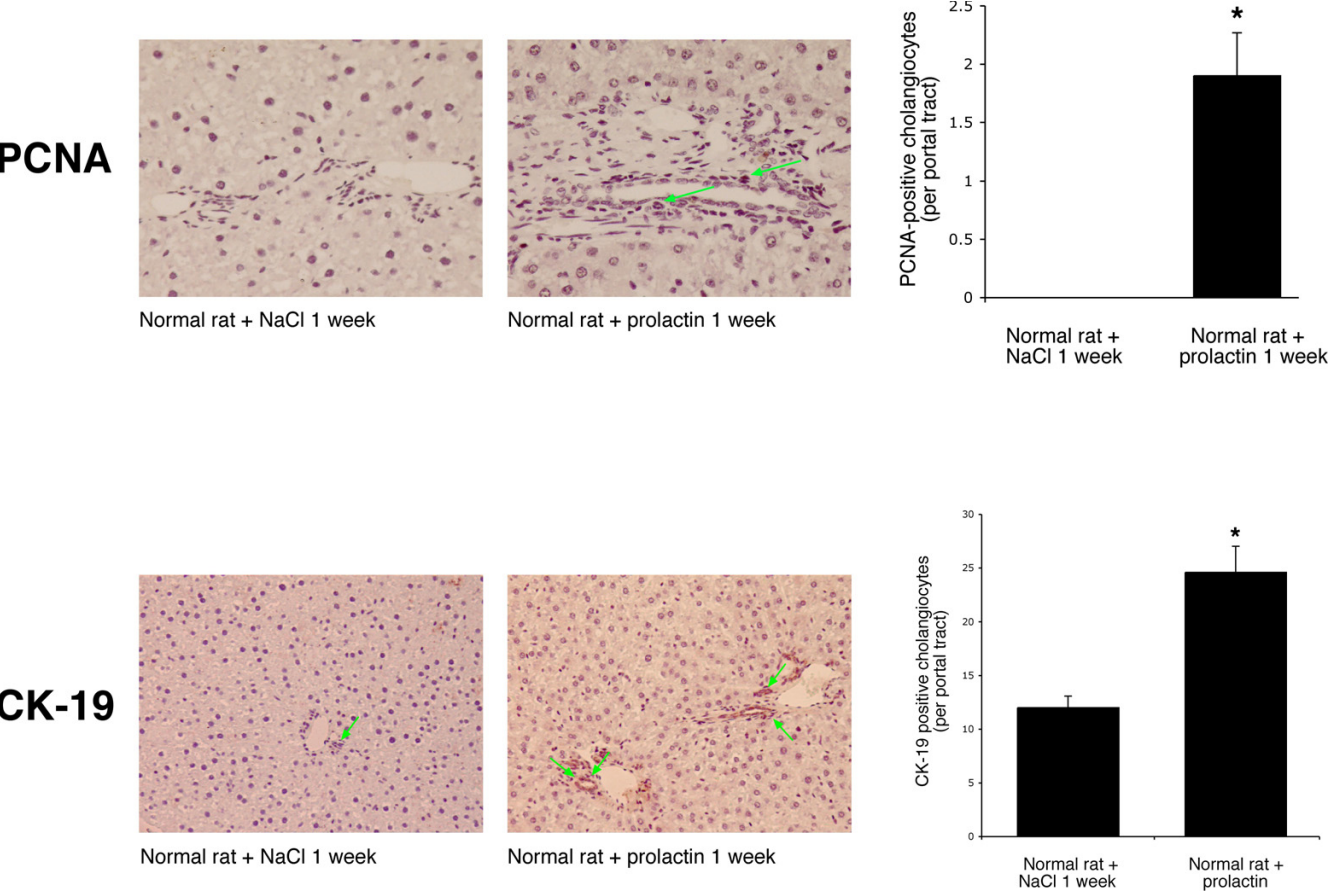
Measurement of the number of [top panel] PCNA- and [lower panel] CK-19-positive cholangiocytes in liver sections (5 μm, 3 slides analyzed per group) and [c] PCNA protein expression in purified female cholangiocytes from NaCl- or prolactin-treated rats. Administration of prolactin to normal female rats increased the number of PCNA-positive cholangiocytes (arrows) and CK-19-positive cholangiocytes compared with normal rats treated with NaCl. Orig. magn., ×20 (PCNA) and ×10 (CK-19). Data are mean ± SEM of 5 values obtained from the 3 slides evaluated per each group of animal. * p < 0.05 vs. the corresponding value of NaCl-treated rats.

Chronic *in vivo *administration of prolactin to normal female rats increased prolactin serum levels compared to normal rats treated with NaCl for 1 week (Table 1). H&E staining of paraffin-embedded liver sections demonstrated that there were no significant differences in the degree of portal inflammation, necrosis, apoptosis and lobular damage between NaCl- and prolactin-treated normal female rats (Table 1). Administration of prolactin to normal female rats increased the number of proliferating cellular nuclear antigen (PCNA)- and CK-19-positive cholangiocytes compared with normal rats treated with NaCl (Figure 4). Prolonged administration of prolactin to normal male rats did not change intrahepatic ductal mass (evaluated by γ-GT histochemistry) [30] compared to normal male rats treated with NaCl [1.0 ± 0.2 % volume (normal + prolactin) vs. 1.0 ± 0.2 % volume (normal + NaCl); not significantly different].

**Table 1:** Evaluation of prolactin serum levels, inflammation, necrosis, lobular damage and apoptosis in liver sections after *in vivo *administration of: (i) NaCl or prolactin to normal rats for 1 week; or (ii) anti-prolactin antibody or non-immune serum to BDL (immediately following BDL) rats for 1 week.

**Treatment**	**Normal rats + NaCl for 1 week**	**Normal rats + PRL for 1 week**	**BDL rats + non-immune serum for 1 week**	**BDL rats + anti-PRL antibody for 1 week**
Prolactin serum levels (ng/ml)	6.6 ± 0.16	98.4 ± 1.5*	63.4 ± 1.0	14.0 ± 0.2*
Inflammation	0 ± 0	0.4 ± 0.2^ns^	1.6 ± 0.2	0.8 ± 0.2*
Necrosis	0 ± 0	0.42 ± 0.2^ns^	1.0 ± 0.0	0.6 ± 0.2*
Lobular damage	0.06 ± 0.06	0.26 ± 0.11^ns^	1.8 ± 0.14	0.9 ± 0.06*
Cholangiocyte Apoptosis	0 ± 0	0.2 ± 0.2^ns^	0.6 ± 0.2	0.8 ± 0.2^ns^

Inflammation, necrosis, lobular damage and apoptosis were evaluated in paraffin embedded liver sections (5 μm) stained with hematoxylin and eosin. *In vivo *administration of prolactin to normal rats for 1 week increased prolactin serum levels but did not alter liver inflammation, necrosis, lobular damage or apoptosis compared to NaCl-treated normal female rats. The administration of anti-prolactin antibody to BDL female rats decreased prolactin serum levels and ameliorated portal inflammation, necrosis and lobular damage compared to BDL rats treated with non-immune serum for 1 week. Data on prolactin serum levels are mean ± SEM of 3 samples from 3 different rats. Data (mean ± SEM) related to the measurement of inflammation, necrosis, lobular damage and cholangiocyte apoptosis are obtained from the analysis of 3 slides per portal tract. *p < 0.05 vs. its corresponding value from BDL rats treated with non-immune serum.

### Effect of prolactin on [Ca^2+^]_i _levels and phosphorylation of Ca^2+^-dependent PKC isoforms in normal female cholangiocytes

**Figure 5:**
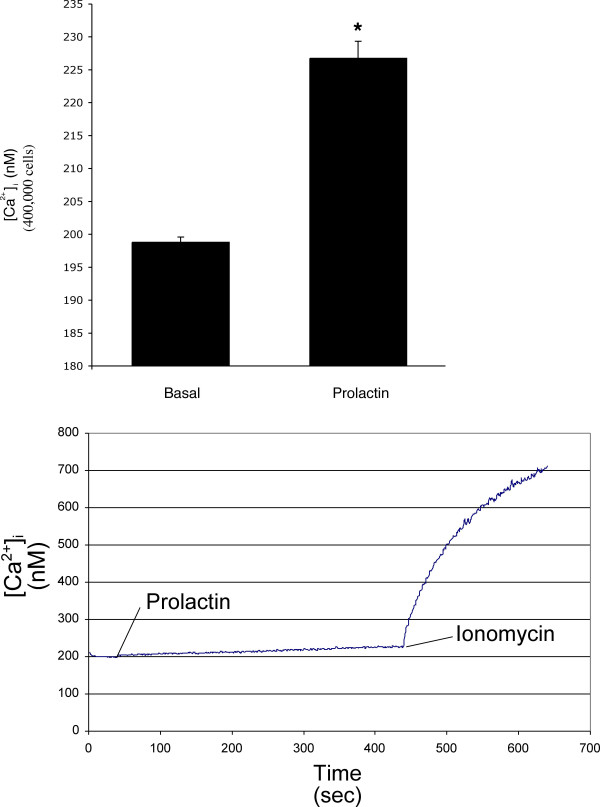
Determination of [Ca^2+^]_i _levels in female normal rat cholangiocytes treated with 0.2% BSA or 100 nM prolactin. Prolactin induced a sustained increase in [Ca^2+^]_i _levels compared with cholangiocytes treated with 0.2% BSA [top panel]. Data are mean ± SEM of 4 experiments. * p < 0.05 vs. the corresponding basal values. [lower panel] A calcium tracing, which is the average of three independent measurements, is shown in shown. As the tracing shows there is no change in fluorescence during the basal measurement period that demonstrates that the cells do not leak as influx of extracellular calcium would alter fluorescence. Cholangiocyte responsiveness to the Ca^2+ ^ionophore, ionomycin, is shown.

Prolactin induced a sustained increase in [Ca^2+^]_i _levels in normal female cholangiocytes compared with cholangiocytes treated with 0.2% BSA (Figure 5, top panel). A calcium tracing, which is the average of three independent measurements, is shown in Figure 5 (lower panel). As the tracing shows there is no change in fluorescence during the basal measurement period that demonstrates that the cells do not leak as influx of extracellular calcium would alter fluorescence (Figure 5, lower panel). Cholangiocyte responsiveness to the Ca^2+ ^ionophore, ionomycin [31], is shown in Figure 5 (lower panel).

**Figure 6:**
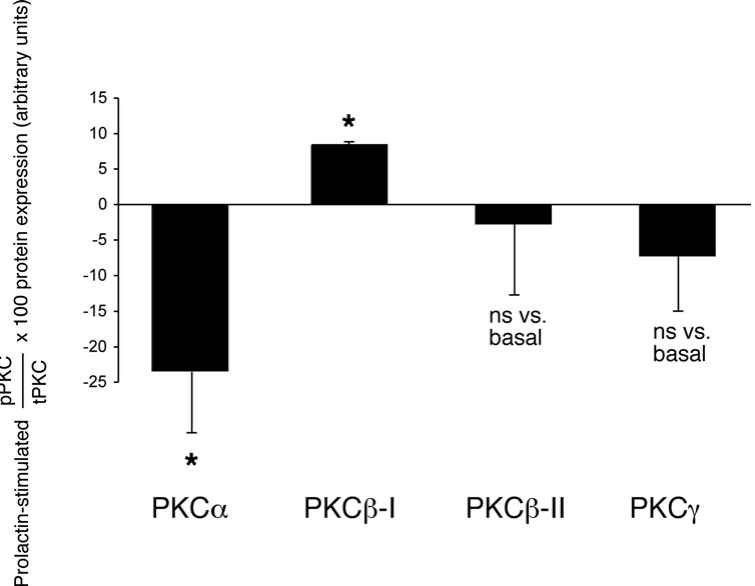
*In vitro *effect of prolactin on the phosphorylation of Ca^2+^-dependent PKC isoforms. Immunoblots for PKC-α, PKC-β-I, PKC-β-II and PKC-γ in normal female cholangiocytes stimulated for 90 minutes at 37°C with 0.2% BSA (basal value) or prolactin (100 nM) with 0.2% BSA. When cholangiocytes were treated with prolactin, there was an increase in the phosphorylation of PKCβ-I and a marked decrease in PKCα phosphorylation; no significant changes in the phosphorylation of PKCβ-II and PKCγ were observed in normal female cholangiocytes treated with prolactin or 0.2% BSA. Data are mean ± SEM of 3 experiments. * p < 0.05 vs. corresponding basal values. PKC = protein kinase C.

When purified female cholangiocytes were treated with prolactin, there was an increase in the phosphorylation of PKCβ-I and a decrease in PKCα phosphorylation (Figure 6); no significant changes in the phosphorylation of PKCβ-II and PKCγ were observed in normal female cholangiocytes treated with prolactin (Figure 6).

### Effect of *in vivo *administration of anti-prolactin antibody on cholangiocyte proliferation of BDL rats

#### Cholangiocytes express prolactin

**Figure 7:**
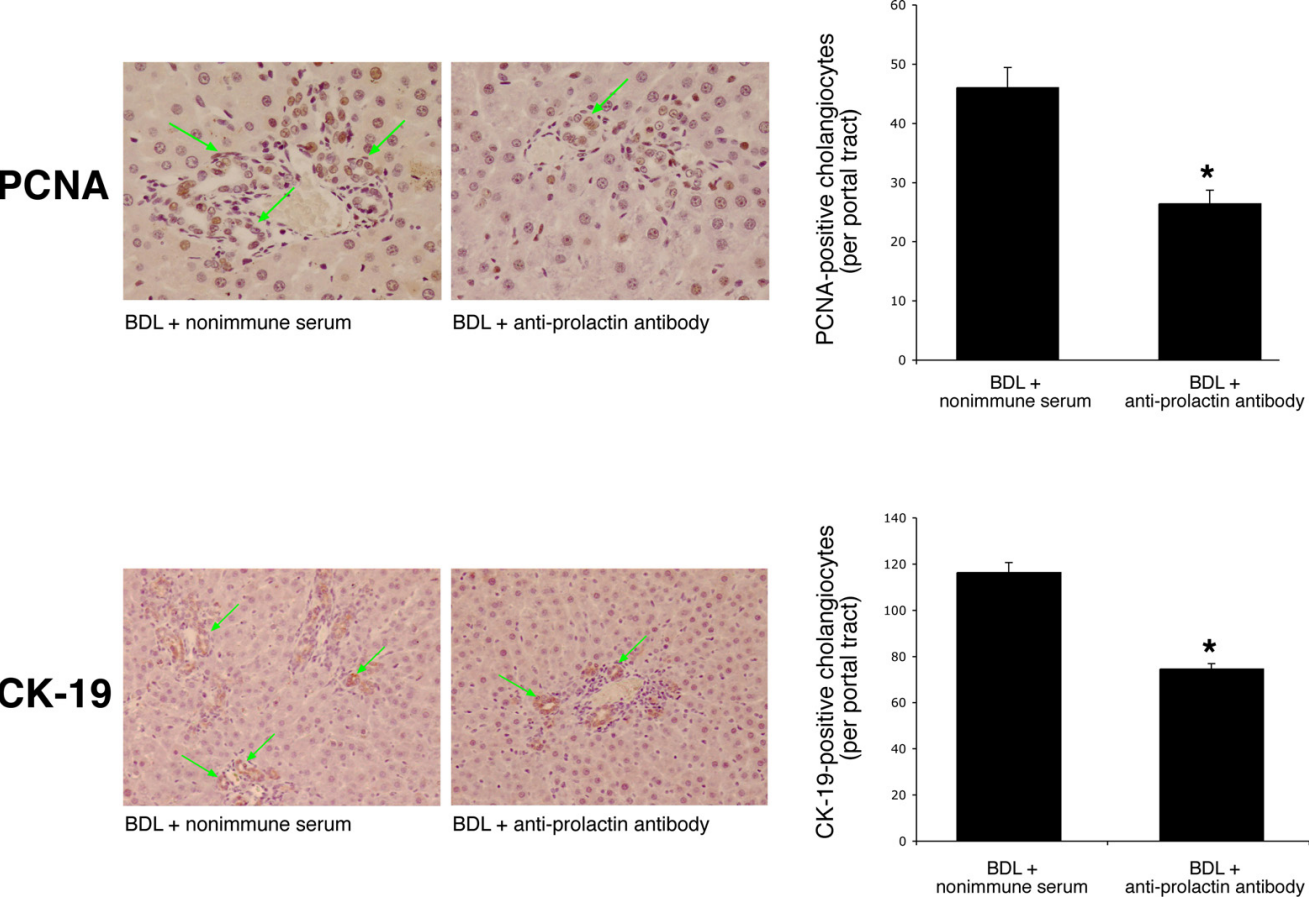
Administration of anti-prolactin antibody to female BDL rats decreased the number of [top panel] PCNA-positive cholangiocytes and [lower panel] CK-19-positive cholangiocytes compared to cholangiocytes from BDL female rats treated with non-immune serum. Orig. magn., ×20 (PCNA) and ×10 (CK-19). Data are mean ± SEM of 5 values obtained from the 3 slides evaluated per each group of animal. * p < 0.05 vs. the corresponding value of BDL rats treated with non-immune serum.

The administration of anti-prolactin antibody to BDL female rats decreased prolactin serum levels and ameliorates portal inflammation, necrosis and lobular damage compared to BDL rats treated with non-immune serum for 1 week (Table 1). Administration of anti-prolactin antibody to BDL female rats decreased the number of PCNA- and CK-19-positive positive cholangiocytes compared to liver sections from BDL rats treated with non-immune serum (Figure 7). Prolonged administration of anti-prolactin antibody to BDL male rats did not change intrahepatic ductal mass (evaluated by γ-GT histochemistry) [30] compared to BDL male rats treated non-immune serum [4.5 ± 1.0 % volume (BDL + anti-prolactin antibody) vs. 3.7 ± 0.8 % volume (BDL + non-immune serum); not significantly different].

**Figure 8:**
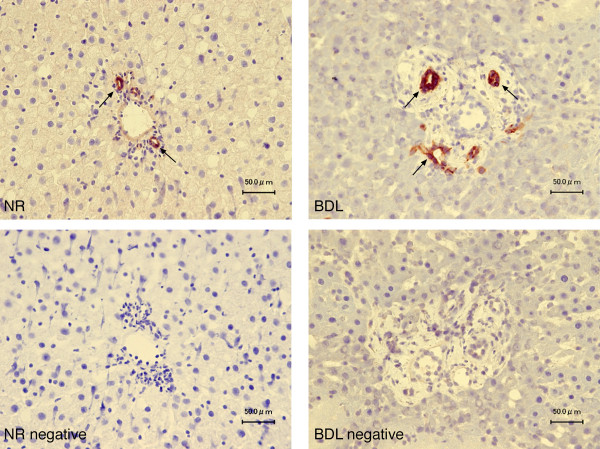
Immunohistochemistry for prolactin in liver sections of normal and BDL female rats shows that intrahepatic bile ducts express the protein for prolactin (arrows). Bar = 50 μm.

**Figure 9:**
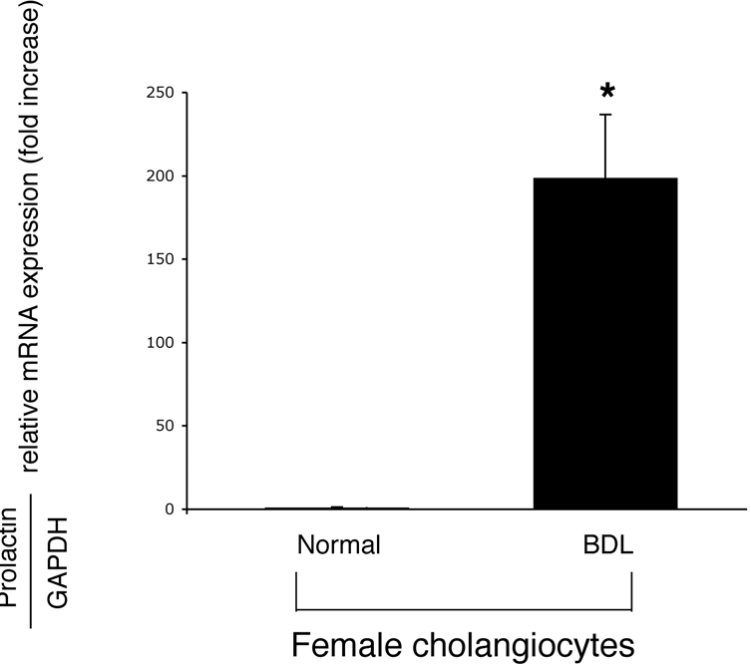
Real time PCR for prolactin mRNA in total RNA from normal and BDL female cholangiocytes. We demonstrated that: (i) female normal cholangiocytes express prolactin mRNA at low levels; and (ii) following BDL, prolactin mRNA markedly increased in female cholangiocytes. Data are mean ± SEM of 3 experiments. *p < 0.05 vs. relative expression of prolactin receptor of normal female cholangiocytes.

By immunohistochemistry in liver sections, bile ducts from normal and BDL female rats express the protein for prolactin (Figure 8, arrows). No staining was seen when a consecutive liver section of the same field was incubated with non-immune serum (Figure 8). By real time PCR, we have demonstrated that: (i) female normal cholangiocytes express prolactin mRNA at low levels (Figure 9); (ii) following BDL, prolactin mRNA markedly increased in female cholangiocytes (Figure 9); and (iii) primary cultures of normal and BDL female cholangiocytes secrete prolactin [14.0 ± 1.0 ng/ml (normal cholangiocytes); and 11.0 ± 2.2 ng/ml (BDL cholangiocytes); n = 7).

## Discussion

Our study demonstrates that prolactin regulates the growth of female cholangiocytes presumably by an autocrine mechanism. We first demonstrated in liver sections that cholangiocytes from normal and BDL female and male rats express prolactin receptors. By real time PCR: (i) normal female cholangiocytes expressed both the short and long form of prolactin receptor mRNA; and (ii) following BDL, the expression of the short and long form of prolactin receptors increased in female cholangiocytes. Our data on cholangiocyte prolactin receptor expression are slightly different to those of previous studies in albino mongrel rats [23] showing that: (i) normal isolated intrahepatic bile duct units (IBDU) predominantly express the message for the long form of the prolactin receptor, whereas the expression of the short form of the prolactin receptor is very low or absent [23]; and (ii) following BDL, the genetic expression of the long form markedly increases in IBDU whereas the short form of prolactin receptor slightly increased in IBDU [23]. The slight difference between these data is presumably due to the different strain of rats used in our studies (female 344 Fischer) and the other studies (albino mongrel) [23]. Prolactin receptors are expressed by rat hepatocytes in the sinusoidal domain of cellular membranes and in perinuclear areas [32]. Prolactin receptors are also expressed by human hepatocytes of patients with obstructive jaundice of different etiology, but prolactin receptor expression is lower in hepatocytes compared to human cholangiocytes [21]. Although these previous studies have shown that cholangiocytes express prolactin receptors [21,23], no information exists on the role of prolactin in the regulation of cholangiocyte hyperplasia.

We next performed *in vivo *studies and demonstrated that the administration of: (i) prolactin to normal female rats induces cholangiocyte hyperplasia devoid of portal inflammation and hepatic damage; and (ii) anti-prolactin antibody to BDL female rats decreases cholangiocyte proliferation and ameliorates portal inflammation and hepatic damage. The most likely explanation why prolactin increased cholangiocyte growth but not portal inflammation and hepatic damage in normal rats is that prolactin induces cholangiocyte hyperplasia as a direct effect and not as a consequence of obstructive cholestasis (i.e., BDL), a pathological condition associated with increased portal inflammation [33,34]. In support of our findings, a number of studies have shown that certain bile acids, vascular endothelial growth factor and forskolin induce cholangiocyte hyperplasia devoid of apoptosis, necrosis, hepatic damage or portal inflammation [31,35,36]. However, in BDL, which is accompanied by an inflammatory response along with cholestasis [33,37,38], the blocking of prolactin with an antibody reduces hepatic damage and cholangiocyte proliferation along with suppression of some inflammatory responses.

Concomitant with enhanced ductal hyperplasia, there was increased prolactin serum levels in normal female rats treated with prolactin compared to NaCl treated rats. In BDL female rats, the serum levels of prolactin increased approximately 15-fold as compared to the levels of normal female rats. Moreover, the administration of anti-prolactin antibody to BDL rats reduces not only cholangiocyte proliferation but also prolactin serum levels. We suggest that changes in prolactin serum levels may be important in the regulation of cholangiocyte growth in chronic cholestatic liver diseases.

In different cell types, prolactin effects are mediated by an increase in Ca^2+ ^levels and PKC activation [11,39]. Thus, we evaluated the role of the Ca^2+^/PKC signaling pathway in prolactin regulation of cholangiocyte hyperplasia. Our results show that [Ca^2+^]_i _levels are increased in normal female cholangiocytes after *in vitro *prolactin stimulation as compared to cholangiocytes stimulated with BSA. From our previous experience with purified cholangiocytes and cholangiocarcinoma cell lines we do not expect a traditional calcium spike [24,26,29,40,41]. In our previous studies [24,26,29,40,41], we demonstrated that the Ca^2+ ^dynamics of cholangiocytes are in general slower and not characterized by a Ca^2+ ^spike [24,26,29,40,41]. The method that we currently employ results in Ca^2+ ^measurements [29,31], which are an average signal of 400,000 cells rather than typical single cell measurements [24,26,40]. This approach gives us similar data [29,31] to that obtained with measurements, which were made in single cells loaded with Fluo-3AM [24,26,40]. Since the measurements are taken in a large number of cells the cellular response to prolactin is not synchronized in the studies. Thus, any peak(s) present will be muted and spread out over time, which is a factor contributing to the slow drift observed. In support of our finding, Ducret *et al*. demonstrated a similar slow response calcium wave due to prolactin in glia cells [42].

We next evaluated if prolactin stimulation of cholangiocyte proliferation was coupled with phosphorylation/dephosphorylation of specific Ca^2+^-dependent PKC isoforms. Our finding that prolactin stimulation of normal female cholangiocyte proliferation is associated with concomitant increased (PKCβ-I) and decreased (PKCα) PKC phosphorylation suggests that a counterbalancing system between PCKβ isoform and PKCα may regulate cholangiocyte proliferation following prolactin stimulation. In support of this concept, while enhanced phosphorylation of PKCβ-II mediates the activation of secretin-stimulated ductal secretion [29] (a functional marker of cholangiocyte growth) [1,2,4,6,25,43] of BDL rats, increased phosphorylation of PKCα (which is inversely related to cholangiocyte growth) is associated with reduced cholangiocyte growth [24,25,40]. In agreement with the view, in hematopoietic [44] and glioma [45] cells, the activation of PKCβ-I and β II isoforms leads to an increase in cell proliferation. Furthermore, in intestinal cell lines the overexpression and/or activation of PKCα decreases cell growth and tumorigenicity [46]. Taken together, our studies show that the Ca^2+^/PKCβ-I and α signaling pathway is one of the players involved in prolactin regulation of cholangiocyte proliferation, but did not evaluate if other pathways (e.g., JAK/STAT and 17-hydroxy-steroid dehydrogenase) modulate prolactin effects on cholangiocyte growth. Also, our studies do not establish which isoform (short or long) of the prolactin receptor mediates the effects of prolactin on cholangiocyte growth. However, based upon previous studies showing that the long form of prolactin receptor mediates increases in [Ca^2+^]_i _in other cells [18,19,47], we propose that the long form of the prolactin receptor may be the major player in prolactin modulation of cholangiocyte growth.

Next, we demonstrated that normal and BDL female cholangiocytes express the message and protein for prolactin and secrete prolactin in primary cultures. The reason why prolactin secretion is similar in normal and BDL female cholangiocytes (although prolactin message expression increases in BDL cholangiocytes) may be due to post-transcriptional events (e.g., message stability/degradation) affecting the translation of the prolactin message. On the basis of these findings, although our studies do not provide direct evidence for the following concept, we propose that prolactin may regulate cholangiocyte proliferation by an autocrine mechanism (in addition to a paracrine pathway). In agreement with the latter concept, a number of cells including mammary epithelial cells, fibroblasts, and cancer cell lines [12,15] secrete prolactin, thus regulating their functions. Furthermore, in support of the concept that prolactin regulates cholangiocyte proliferation by an autocrine mechanism, we have previously shown that cholangiocytes express/secrete neurotrophins [33], vascular endothelial growth factor [31] and serotonin [48], thus regulating intrahepatic ductal mass by an autocrine mechanism [31,33,48].

## Conclusion

In summary this study has shown that: (i) cholangiocytes express both isoforms (long and short) of the receptor for prolactin; (ii) prolactin has a trophic effect on the growth of normal female cholangiocytes by phosphorylation of PKCβ-I and dephosphorylation of PKCα; and (iii) cholangiocytes express the message/protein for and secrete prolactin, findings suggesting that prolactin participates, by an autocrine mechanism, in the modulation of cholangiocyte proliferation. Prolactin may be an important therapeutic approach for the management of cholangiopathies.

## Methods

### Materials

Reagents were purchased from Sigma Chemical (St Louis, MO) unless otherwise indicated. The RIA kit for the measurement of prolactin levels in serum and cholangiocyte supernatant was purchased from GE Healthcare Bio-Sciences Corp. (Piscataway, NJ). The monoclonal mouse antibody against PCNA was purchased from DAKO (Kyoto, Japan). PCNA is a nonhistone nuclear protein that plays an important role in DNA replication and cellular proliferation by interacting with DNA polymerase-delta) [49]. The substrate for γ-glutamyl transpeptidase (γ-GT), N (γ-L-glutamyl)-4-methoxy-2-naphthylamide was purchased from Polysciences (Warrington, PA). Antibodies against prolactin and the Ca^2+^-dependent PKC isoforms (α, β-I, β-II and γ) were purchased from Santa Cruz Biotechnology Inc. (Santa Cruz, CA). The sheep polyclonal (ab35349) antibody recognizing the prolactin receptor (used for the immunohistochemical and immunofluorescent evaluation of prolactin receptor in liver sections) was purchased from Abcam (Cambridge, UK). This antibody does not distinguish between the short or long form of the prolactin receptor. The RNeasy Mini Kit to extract total RNA from purified cholangiocytes was purchased from Qiagen Inc, Valencia, CA.

### Experimental model

Female or male Fisher rats (150–175 gm) were purchased from Charles River (Wilmington, MA) and maintained in a temperature-controlled environment (20–22°C) with a 12:12-hour light-dark cycle. Rats were fed *ad libitum *standard chow and had free access to drinking water. To evaluate the *in vivo *effect of prolactin on cholangiocyte growth, normal female or male rats were injected twice per day with NaCl or ovine prolactin (420 μg/rat per day, a dose similar to that used in other studies in rodents) [50] for 1 week. We evaluated the effect of the administration of anti-prolactin antibody on cholangiocyte proliferation of BDL female or male rats. Immediately after BDL [43], rats received 200 μL of non-immune serum or polyclonal neutralizing prolactin antibody (400 pg/dose, intraperitoneally every day) [51] for 7 days. Before each experimental procedure, animals were injected with sodium pentobarbital (50 mg/kg weight, IP). Study protocols were performed in compliance with the institutional guidelines.

### Purification of cholangiocytes

Cholangiocytes were isolated by immunoaffinity separation [1,2,25,52,53], using a mouse monoclonal antibody (IgM, provided by Dr. R. Faris, Brown University, Providence, RI) that recognizes an unidentified antigen expressed by all intrahepatic rat cholangiocytes [52]. The purity of cholangiocytes was evaluated by γ-GT histochemistry [30]. Cell viability (by trypan blue exclusion) ranged from 95 to 98%.

### Expression of prolactin receptors in cholangiocytes

For immunohistochemistry, after deparaffination of liver sections (5 μm thick; 3 slides analyzed per group), endogenous peroxidase activity was quenched for 5 minutes with methanol-peroxide solution (0.3% hydrogen peroxide solution, Santoku Chemical Industries, Tokyo, Japan) in 80% methanol (WAKO, Osaka, Japan). Sections were hydrated in graded alcohol and rinsed in 1× phosphate-buffered saline (1× PBS, pH 7.4) before applying the antibody specific for prolactin receptor (diluted 1:400) or non-immune serum at 4°C overnight. After rinsing with PBS, Histofine Simple Stain Rat (Multi) (Nichirei, Tokyo, Japan) was added as secondary antibody for 1 hour at room temperature. Nuclear counterstaining was performed using hematoxylin for light microscopy after detecting reactions with VECTOR NovaRED (Vector Laboratories, Inc., Burlingame, CA). Following staining, sections were observed with the light microscope ECLIPSE E600 (Nikon, Tokyo, Japan).

For immunofluorescence, frozen liver sections (20 μm thick; n = 3 per each group of animals) were fixed in 4% paraformaldehyde (in 1× PBS) for 10 minutes, followed by tissue permeabilization in PBST (1× PBS with 0.2% triton X-100). Non-specific protein binding was blocked by 5% normal goat serum. Following incubation with a primary antibody against prolactin receptor (raised in sheep, 1:5; Abcam) or non-immune goat serum (negative control), together with an anti CK-19 antibody (raised in mouse, 1:50; Vision Biosystems Inc, Norwell, MA), sections were rinsed with 1× PBS and subsequently incubated with Cy2-conjugated anti-mouse and Cy3-conjugated anti-sheep antibodies (both diluted at 1:50) (Jackson Immunochemicals, West Grove, PA). Following staining, sections were observed either with the light microscope ECLIPSE E600 (Nikon, Kawasaki, Japan) or fluorescence microscope DMRXA/HC (Leica, Tokyo, Japan).

We first performed RT-PCR for the short and long form of prolactin receptor to determine that cholangiocytes express the expected molecular weight band for these two receptor isoforms and GAPDH, the housekeeping gene [1]. Thereafter, the same primers were used to determine (by real time PCR) the quantitative expression of short and long prolactin receptors in total RNA (0.75 μg) from normal and BDL female cholangiocytes. The primers (from Integrated DNA Technologies, Coralville, IA) were designed according to the sequences for the short (NCBI Genbank Accession No. NM 012630) [23] and long prolactin receptor mRNAs (NCBI Genbank Accession No. NM 001034111) [23]. The 5' primer, 5'-CAAATGGGAAGCAGTTCCTC-3' (common) was designed to a region that is homologous to both the short and long forms. The short form 3' primer, 5'-AGGAAGGGCCAGGTACAGAT-3' (short), was taken from a sequence region of the short form mRNA that is non-homologous to long form mRNA. The long form 3' primer, 5'-GGGGTTCCTCACACTTTTCA-3' (long), was taken from a sequence region of the long form mRNA that is non-homologous to the short form mRNA. The primers for GAPDH (sense 5'-GTGACTTCAACAGCAACTCCCATTC-3' and antisense 5'-GTTATGGGGTCTGGGATGGAATTGTG-3', 294 bp) were based on the rat GAPDH sequence [54]. Standard RT-PCR conditions were used with 1 μg of total mRNA (35 step cycles: 30 sec at 94°C, 30 sec at 59°C and 45 sec at 72°C). The PCR samples for prolactin receptor short (582 bp) and long (781 bp) forms were run on agarose gels, the bands excised and removed from the gel with the Qiaquick Gel Extraction Kit (Qiagen, Valencia, CA). The purified fragments were sequenced by Davis Sequencing (Davis, CA).

We used the RT^2 ^Real-Time assay from SuperArray (Frederick, MD) to evaluate the expression of the short and long form of prolactin receptor mRNA in female cholangiocytes from normal and BDL rats. RNA was reverse transcribed using the Reaction Ready™ First Strand cDNA synthesis kit (SuperArray). As described previously [55], 1 μl of the cDNA template was added to 12.5 μl of master mix, 10.5 μl of DI water and 1 μl of RT^2 ^PCR rat primers (SuperArray, Frederick, MD) designed specifically for the messages for short and long prolactin receptor (Integrated DNA Technologies, Coralville, IA) and GAPDH (SuperArray). A ΔΔCT analysis was performed using the normal pooled cholangiocytes as the control sample. Data was expressed as relative mRNA levels ± SEM of short or long prolactin receptor to GAPDH ratio (n = 3). To confirm the presence of one PCR product (by real time PCR analysis), we performed a dissociation analysis and observed the presence of only one peak for all primers.

### Evaluation of portal inflammation, lobular damage, necrosis and cholangiocyte apoptosis and proliferation

We evaluated the effect of *in vivo *administration of: (i) NaCl or prolactin to normal rats; and (ii) anti-prolactin antibody or non-immune serum to BDL rats on portal inflammation, lobular damage, necrosis and cholangiocyte apoptosis and proliferation. Paraffin embedded liver sections (5 μm, 3 sections analyzed per group) were stained with hematoxylin and eosin (H&E) before determining lobular damage, necrosis and the degree of portal inflammation as previously described by us [41]. Terminal deoxynucleotidyl transferase biotin-dUTP nick end labeling (TUNEL) analysis was performed using a commercially available apoptosis detection kit (TACS™ TdT kit, R&D systems, Minneapolis, MN). Following the selected staining, sections (5 μm, 3 slides analyzed per group) were evaluated in coded fashion with the microscope ECLIPSE E600 (Nikon Eclipse, Tokyo, Japan). Two hundred cells per slide were counted in a coded fashion in ten non-overlapping fields.

Cholangiocyte proliferation was evaluated by quantitative determination of the number of PCNA- and CK-19-positive cholangiocytes in liver sections from the selected groups of animals. Immunohistochemistry for PCNA or CK-19 was performed in paraffin-embedded sections (5 μm, 3 slides analyzed for each group) as described [2,5]. Sections were counterstained with hematoxylin and examined with the ECLIPSE E600 microscope (Nikon Eclipse, Tokyo, Japan). Over 100 cholangiocytes were counted in a random, blinded fashion in three different fields for each section. Data were expressed as number of PCNA- or CK-19-positive cholangiocytes per each 100 cholangiocytes.

### Evaluation of the intracellular signaling pathway by which prolactin regulates normal cholangiocyte proliferation

We performed *in vitro *experiments in normal female cholangiocytes to demonstrate that: (i) prolactin increases intracellular Ca^2+ ^levels; and (ii) specific Ca^2+^-dependent PKC isoforms play a role in prolactin regulation of cholangiocyte proliferation. Following purification, cholangiocytes were incubated for 1 hour at 37°C to regenerate membrane proteins [1,6,24-26,29,56] damaged by proteolytic enzymes during isolation [52] prior to loading with Fluo-3AM before [Ca^2+^]_i _measurements. A number of studies have demonstrated that following the incubation time of 1 hour at 37°C cholangiocytes are functionally responsive [1,26,29,33,52,56]. [Ca^2+^]_i _fluorescence measurements in cholangiocytes were performed using fluo-3 AM (Molecular Probes, Eugene, Oregon) and a Fluoroskan Ascent FL (ThermoLabsystems, Helsinki, Finland) microplate reader equipped with three injectors [29,57]. Cholangiocytes (4 × 10^4 ^per well) were loaded for 1 hour at room temperature with 5 μM of fluo-3 AM in Tyrode's salt solution (TSS, 137 mM NaCl, 2.7 mM KCl, 1 mM MgCl_2_, 0.2 mM NaH_2_PO_4_, 12 mM NaHCO_3 _and 5.5 mM glucose) with O.1% Pluronic F-127 (Molecular Probes, Eugene, Oregon). After washes with TSS, the loaded cells were added to a 96 well black microplate. The baseline fluorescence was measured 50 times after 1 second at 2-second intervals. TSS alone or prolactin (100 nM) dissolved in buffer was injected sequentially into separate wells, and the fluorescence intensity was measured at 538 nm for 3 minutes at 1-second intervals. The excitation wavelength was 485 nm. [Ca^2+^]_i _concentration was calculated as follows: [Ca^2+^]_i _= K_d_(F-F_min_)/(F_max_-F). F_max _refers to fluorescence intensity measured after permeabilization of the cells with 1% NP-40. Then, 0.1 M EGTA was added to chelate Ca^2+ ^and minimum fluorescence intensity (F_min_) was obtained. Ionomycin (10 μM) was utilized at the end of each calcium determination to ensure cholangiocyte responsiveness.

Purified normal female cholangiocytes were stimulated for 90 minutes [25-27] at 37°C with 0.2% BSA (basal) or prolactin (100 nM) with 0.2% BSA, and analyzed for protein expression of the phosphorylated form (expressed as ratio to total protein expression of the corresponding PKC isoform) of the selected PKC isoform by immunoblots. The intensity of the bands was determined by scanning video densitometry using the phospho-imager, Storm 860, Amersham Biosciences (Piscataway, NJ) using the ImageQuant TLV 2003.02 (Little Chalfont, Buckinghamshire UK).

### Evaluation of expression and secretion of prolactin by cholangiocytes

We performed immunohistochemistry (in liver sections) to evaluate if female cholangiocytes from normal and BDL rats express the protein for prolactin. Immunohistochemistry for prolactin in liver sections was performed in the same manner as described for prolactin receptor staining except using a goat polyclonal anti-prolactin as primary antibody.

We evaluated by real time PCR the quantitative expression of prolactin in total RNA (0.75 μg) from purified cholangiocytes from normal and BDL female and male rats. Real time PCR for prolactin was performed as described above for prolactin receptor with the exception of the primers that were purchased from SuperArray.

To determine the amount of prolactin secreted, purified cholangiocytes from normal and BDL female rats were incubated at 37°C for zero and six hours. Thereafter, cells were centrifuged at 1,500 rpm for 10 minutes at 4°C, the supernatant transferred to a clean tube and stored at -70°C before analysis for prolactin levels by RIA by commercially available kits (GE Healthcare Bio-Sciences Corp.).

### Statistical analysis

We expressed all data as mean ± SEM. The differences between groups were analyzed by Student's t-test if two groups were analyzed or analysis of variance (ANOVA) if more than two groups were analyzed (assuming p < 0.05 as statistical difference between the analyzed groups).

## Abbreviations

BSA = bovine serum albumin; BDL = bile duct ligation; CK-19 = cytokeratin-19; GAPDH = glyceraldehyde-3-phosphate dehydrogenase; γ-GT = γ-glutamyl transpeptidase; MAPK = mitogen-activated protein kinase; PCNA = proliferating cellular nuclear antigen; PBC = primary biliary cirrhosis; PKC = protein kinase C.

## Authors' contributions

ST has made substantial contributions to the conception and writing of the study.

SG has measured intracellular Ca^2+ ^levels and made substantial contribution to the design and writing of the study.

HF has performed RIA analysis, real time PCR and made substantial contribution to the design and writing of the study.

SD has performed the immunofluorescence studies and made substantial contribution to the design and writing of the study.

YU has performed the immunohistochemical and morphometric analyses in liver sections.

DA has made substantial contribution to the conception and writing of the study.

LM has made substantial contribution to the conception and writing of the study.

MM has made substantial contribution to the conception and writing of the study.

GF has made substantial contribution to the conception and writing of the study.

JV has performed RIA analysis and stimulated all the purified cholangiocytes with the selected agonists/antagonists.

SV has performed all the immunoblots.

BV has performed all the cholangiocyte isolations.

IP-YL performed RNA extraction and RT-PCR analysis.

VH-YL performed RNA extraction and RT-PCR analysis.

EG has performed the immunohistochemical analyses for prolactin and prolactin receptor made substantial contribution to the editing of the manuscript.

GC has performed the immunohistochemical analyses for prolactin and prolactin receptor made substantial contribution to the editing of the manuscript.

AB has made substantial contributions to the conception and writing of the study.

GA has made substantial contributions to the conception, design and writing of the study. He oversaw the entire project.

All authors have read and approved the final manuscript.
